# Failing to progress or progressing to fail? Age-for-grade heterogeneity and grade repetition in primary schools in Karonga district, northern Malawi

**DOI:** 10.1016/j.ijedudev.2016.10.004

**Published:** 2017-01

**Authors:** Bindu S. Sunny, Markus Elze, Menard Chihana, Levie Gondwe, Amelia C. Crampin, Masoyaona Munkhondya, Scotch Kondowe, Judith R. Glynn

**Affiliations:** a London School of Hygiene and Tropical Medicine, UK; b Karonga Prevention Study (KPS), Chilumba, Malawi; c District Education Office (DEO), Karonga District, Malawi

**Keywords:** Repetition, School progression, Africa, Over-age, Under-age, Primary school, Risk factors

## Abstract

Timely progression through school is an important measure for school performance, completion and the onset of other life transitions for adolescents. This study examines the risk factors for grade repetition and establishes the extent to which age-for-grade heterogeneity contributes to subsequent grade repetition at early and later stages of school. Using data from a demographic surveillance site in Karonga district, northern Malawi, a cohort of 8174 respondents (ages 5–24 years) in primary school was followed in 2010 and subsequent grade repetition observed in 2011. Grade repetition was more common among those at early (grades 1–3) and later (grades 7–8) stages of school, with little variation by sex. Being under-age or over-age in school has different implications on schooling outcomes, depending on the stage of schooling. After adjusting for other risk factors, boys and girls who were under-age at early stages were at least twice as likely to repeat a grade as those at the official age-for-grade (girls: adjusted OR 2.06 p < 0.01; boys: adjusted OR 2.37 p < 0.01); while those over-age at early stages were about 30% less likely to repeat (girls: adjusted OR 0.65 p < 0.01; boys: adjusted OR 0.72 p < 0.01). Being under/over-age at later grades (4–8) was not associated with subsequent repetition but being over-age was associated with dropout. Other risk factors identified that were associated with repetition included both family-level factors (living away from their mother, having young children in the household, lower paternal education) and school-level factors (higher student-teacher ratio, proportion of female teachers and schools without access to water). Reducing direct and indirect costs of schooling for households; and improving school quality and resources at early stages of school may enable timely progression at early stages for greater retention at later stages.

## 1 Background

Despite global efforts to universalise education, 124 million children worldwide were out of school in 2013 with a growing proportion (50%) of these children living in Sub-Saharan Africa (UIS/UNESCO, 2015, p. 11). While the introduction of free primary education in Malawi led to a significant increase in school enrolments, only 35% manage to complete primary education (World Bank, 2010). Children who drop out of school are not young and may leave school for several reasons, including poor school quality, poor performance or when schooling conflicts with transitions to adulthood (Chimombo et al., 2000; Glynn et al., 2010; Grant and Hallman, 2006; Hunt, 2008; Lloyd et al., 2008; Meekers and Ahmed, 1999; Mensch et al., 1999). In Malawi, primary education is for eight years (grades 1–8), with the official age of entry into school being 6 years. This suggests that those who enter on time and progress uninterruptedly through each grade could complete primary school by the age of 14. However, due to late entry, frequent disruptions and repetitions, children are getting over-age for their grade and may take up to 23 student years to complete eight years of primary education (World Bank, 2010). Age-for-grade heterogeneity (relative age or age “distortion” (Psacharopoulos and Nguyen, 1987) is characterised by children of various ages studying in the same grade in school. Delays in progression result in age heterogeneity within a class, but it is unclear what the extent of age heterogeneity is, and whether being over-age/under-age has an effect on subsequent grade repetition, potentially leading to a vicious circle with students falling further behind the official age-for-grade.

Repetition is an indicator of progress made in school and can result from “academic failure, unsatisfactory progress, insufficient examination marks to advance to the next level of instruction, age, and poor attendance or simply from lack of local educational opportunities”((UNESCO, 2012) p-17). Students in their terminal year of school may “volunteer” to repeat their grade in order to improve their performance in the final exam and increase their chances of securing a place in secondary school; or may choose to repeat a year due to unaffordability of exam fees. Repetition is often practiced in post-colonial Francophone, Anglophone and Lusophone countries in Africa and Asia, and is less common in developed countries (except France and Belgium) where automatic promotion is more prevalent (Ndaruhutse et al., 2008). A global analysis of the patterns of repetition have broadly classified countries to have: a) high repetition (>20%) in early grades, which declines over subsequent grades, till the last grade of lower secondary (like Malawi); b) low repetition in the first grade (<10%), which increases steadily till the last grade of lower secondary; or c) a mix of both, with high repetition in all grades, ranging from 10 to 49% and fluctuations between grades (UNESCO, 2012).

Students who are older at entry have higher repetitions, drop outs and lower completion rates (Wils, 2004), as the productivity of the child and the opportunity cost of being in school increases with age (Cameron, 2005; Majgaard and Mingat, 2012; UNESCO, 2012; Wils et al., 2009). A study on the factors effecting grade repetition in grade 6 in 15 countries in Southern Africa observed that boys from poorer households and under-resourced schools were twice as likely to repeat as those from better off households (Ikeda, 2005). In a study in South Africa, (Branson et al., 2014) those overage for their grade by two or more years were more likely to dropout at later stages. Data from 54 developing countries showed that a higher proportion of female teachers in school reduced repetition for boys and girls, and increased retention especially among girls (cited in (Majgaard and Mingat, 2012). Nutritional status in early years may also be associated with positive school outcomes. A five-cohort study in Brazil, Guatemala, India, Phillipines and South Africa, showed that higher birthweight was associated with a lower risk of grade repetition (Martorell et al., 2010). Recent reviews (Brophy, 2006; Ndaruhutse et al., 2008) have listed a range of individual (low motivation/ability), household (low levels of parental education, household income, participation in household work) and school-level characteristics (low instructional time, differences in mother tongue and language of instruction, high schooling costs, proximity to school, access to sanitation facilities, poor school quality and curriculum relevance) that contribute to grade repetition in school, although there is limited empirical evidence that supports these associations.

While grade repetition is one cause of age-for-grade heterogeneity, the association between age-for-grade heterogeneity and *subsequent* grade repetition is less understood. A descriptive analysis of school performance, using DHS data from 35 countries, showed that over-age students performed better than younger students at early grades, but had higher repetition and dropout at later grades (Wils et al., 2009). In 24 of the 35 countries, including Malawi, almost half of all those in primary school were two or more years over-age for their grade, with those under-age having higher repetition rates, especially in grade 1, while those over-age were at higher risk of dropping out of school.

Our study follows a cohort of primary school students in 2010 in Karonga district, northern Malawi, to understand the risk factors for grade repetition; the extent of age-for-grade heterogeneity in school; and its effects on grade repetition in the following year, after accounting for other individual, household and school-level risk factors.

## 2 Data and methods

### 2.1 Data sources

The dataset for the analysis originates from a Demographic Surveillance System (DSS), collecting data from around 34,000 individuals living in approximately 7000 households in Karonga District, northern Malawi, since 2002. The DSS area is primarily rural, with a majority of the population engaged in agriculture, fishing and petty trading (Crampin et al., 2012). The DSS collects data on births and deaths continuously through key informants within the community, with an annual census. House-to-house surveys following the census include detailed socio-economic, schooling, and demographic profiles of the population. Information on schooling includes current schooling status (in/out of school) and highest educational attainment (grade and level) for individuals above the age of 5. Questions relating to school performance (grade repetition, absenteeism) were asked of those currently in school aged 5–30 years.

Household information includes data on the quality of dwelling construction. A household is defined as a social construct of people who co-reside and acknowledge the same head of household. Interviews were conducted with verbal consent from the household head and individual household members and any reason for non-participation was recorded. GPS locations of individual households and schools (located in and within 10 km of the DSS boundary) were tracked using handheld geographic positioning systems (Garmin Etrex and Garmin Geko 201). Ethics approval for the study was received from the Health Sciences Research Committee, Malawi and the ethics committee of the London School of Hygiene and Tropical Medicine.

### 2.2 Study population

This study focused on respondents between ages 5–24 years, defined by the minimum age for answering the schooling survey; WHO’s definition of “young people”; and the upper age limit observed for those attending primary school. The analysis is restricted to primary school students, as primary schools differ quite markedly from secondary schools, in terms of student profiles (socio-economic status, academic motivation and financial leverage to persist in school), school systems (admission/transition criterion, provision of school infrastructure and resources, funding) and teaching/learning processes (monitoring participation, performance and completion). Enrolment into primary school is free whereas secondary schools are fee-paying and highly selective based on performance at primary level and availability of places.

### 2.3 Dependent and independent variables

Grade repetition as reported in the following year was used as the outcome variable to explicitly delineate the effects of age-for-grade heterogeneity on *subsequent* grade repetition, and distinguish the assumed ordering between exposures and outcome. Respondents were asked about their grade repetition status (“Have you attended your current standard/form before?”) and the number of times they had attended the same grade previously. Age-for-grade, the main explanatory variable, is calculated as the number of years of age a child is ahead/behind in class, based on the official age for a specific grade (i.e. Age-for-grade = Current Age-Current Grade-5). Following UNESCO definitions, respondents were considered over-age if they were two or more years older, and under-age if they were one or more years younger than the lower limit of the official age-for-grade. For example, given the official age of entry in primary schools in Malawi is 6 years, those who are between 6 and 7yrs, > = 8years and < = 5years of age in grade 1, are considered at age, over-age and under-age, respectively (UNESCO Institute for Statistics, 2004). Grades were categorised as early (grades 1–3), mid (grades 4–6) and later stages (grades 7–8) of schooling. School absenteeism data was based on self-reports of the number of days/weeks absent within the last four weeks of being in school.

Socio-economic factors included the highest level of education attained by the father and mother (none/less than primary, at least primary). The number of children in a household below the age of six (which is the official age at school entry), was examined as a possible determinant of school participation for older children who may be responsible for providing childcare at home. Living arrangements of children were also used to establish whether parental presence or absence was associated with participation in school. The quality of dwelling was used as a proxy for long-term household wealth status. Characteristics of houses, including the quality of the roof (plastic, grass/leaves, tiles, iron sheets), floor (mud, concrete, other), glass windows and walls (burnt/unburnt brick, thick/thin mud, concrete), were ranked in ascending order of quality. An overall score was calculated and households were divided into 3 groups (bottom 45%, middle 25% and top 30%) in order of their dwelling score (1 = Worst, 3 = Best). The cut-off points classified households into broader socio-economic groups, in close conjunction with Filmer & Pritchett’s (Filmer and Pritchett, 2001) household classification (i.e., lowest 40% of households as ‘poor’ and highest 20% as ‘rich’) for the construction of wealth indices.

Schools reported in the socio-economic survey were linked data from annual school returns collected by the Karonga District Education Office (DEO) since 2007. Data on school-level characteristics were collated for 24 primary schools, in and around the DSS area (covering 97% of respondents). These included school capacity (number of students, teachers), infrastructure (classrooms, toilets, water and electricity) and performance (enrolment and performance in final exams). Student-teacher ratios were categorised based on the regulation by the Malawian Ministry of Education (MoE) stipulating class sizes to not exceed 60 students(World Bank, 2004). The school performance measure was based on final Primary School Leaving Examination (PSLE), which is an external, national-level terminal exam conducted in grade 8, and is the percentage of students who passed among those entered for the exam. Euclidean distances were calculated from home to school using ArcGIS. Schools which did not have classes up to grade 8 were categorised as incomplete. Access to water (piped/borehole) and electricity (solar/grid) were categorised as binary.

### 2.4 Statistical analysis

The analysis investigates risk factors for grade repetition in 2011 among students in school in 2010 using mixed effects logistic regression models to account for school-level clustering. Variables that independently showed a strong association with grade repetition were selected for the multivariable analysis (Bursac et al., 2008). Age and age at enrolment were excluded from the multivariable analyses as they contribute to the cumulative measure of age-for-grade and grade, which were used instead. Father’s education, number of dependents below the age of six, dwelling score and co-residence status, were identified as potential confounders using bivariate analyses. School absenteeism and school-level characteristics, such as female-teacher ratio, student-teacher ratio and school rank were retained *a priori*. Missingness patterns among the co-variates was investigated for non-random absence of data. Missing data for mother’s education were not missing at random and hence excluded from the multivariable analyses to reduce bias. The model for age-for-grade and grade repetition was *a priori* stratified by gender (interaction of sex with age-for-grade was weak p = 0.76); and stage (early and later) of schooling (interaction of grade with age-for-grade p < 0.01). There were 15 (0.2%) missing observations for the outcome variable (grade repetition). Complete case analysis was conducted for 7877 respondents (96%) for the multivariable analysis.

## 3 Results

**Fig. 1:**
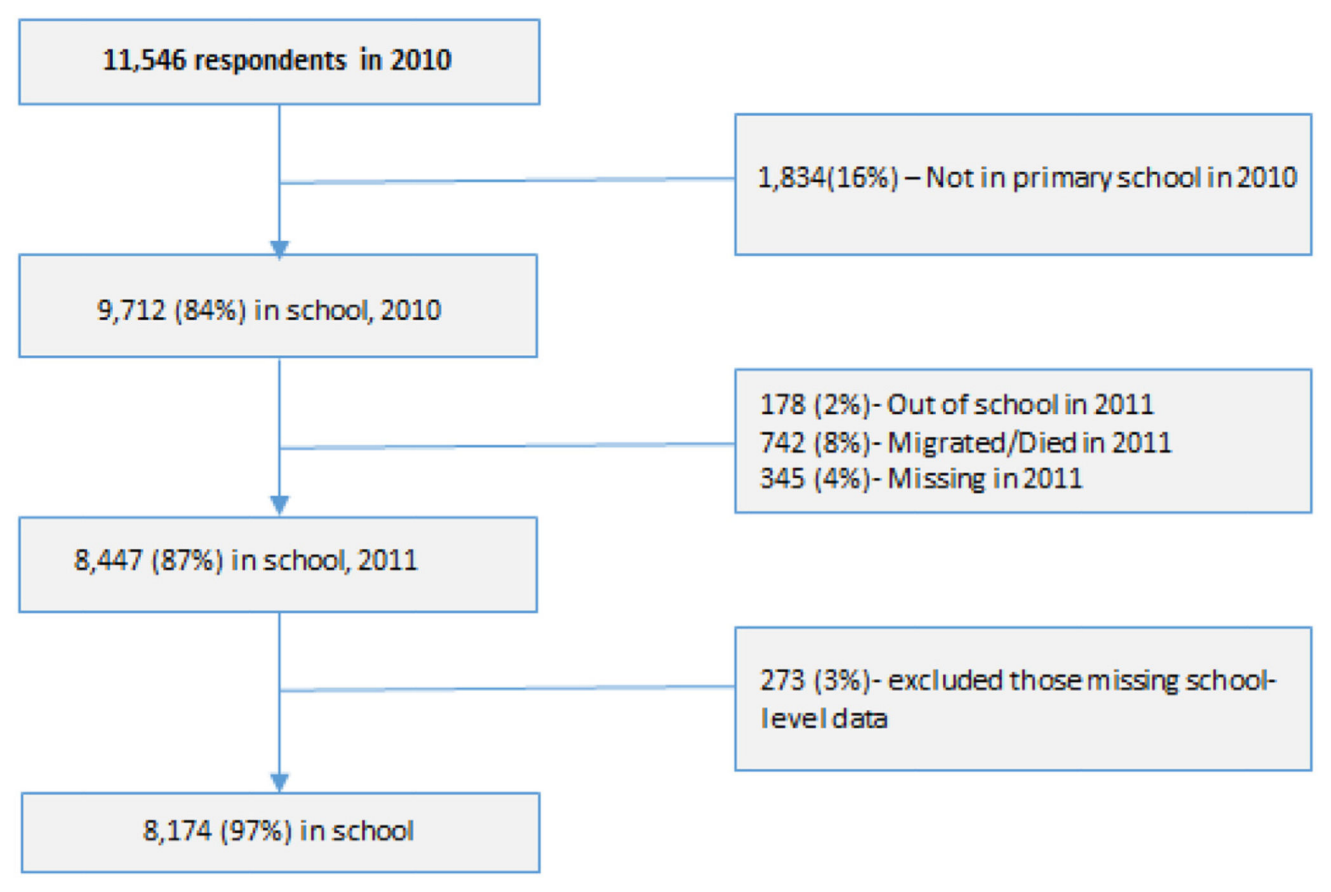
School participation flowchart.

Of the 16,383 individuals eligible to participate (between ages 5 and 24yrs) in the survey in 2010, 109 (0.7%) had left or were not found, 4 (0.02%) had died and 8 refused to participate (0.05%). Of the remaining 16,262 participants (99.2%) who were interviewed, 1168 (7%) were not uniquely identifiable to an individual household in the DSS, as polygamous heads of households or children moving during school-term (Crampin et al., 2012) reported living in multiple households. These individuals were excluded. 11,546 respondents currently/previously in school reported primary as the highest level attended, of whom 9712 (84%) were currently enrolled in primary school in 2010 (Fig. 1).

### 3.1 Characteristics of study population

Table 1 shows the characteristics of these 9712 respondents, and their schooling status in the following year. Most respondents (8447 or 87%) remained in school and were followed-up in 2011 (Fig. 1); 178 had dropped out (2%), 742 had migrated or died (8%), and 345 (4%) were missed. Those who out-migrated/died or were missed had a similar distribution by age, grade and age-for-grade as those in school in the following year. Those who dropped out the following year were older, in higher grades and more likely to be overage for their grade. Being overage by two or more years was significantly associated with dropping out of school (Pearson chi-square p < 0.01, Table 1).

**Fig. 2:**
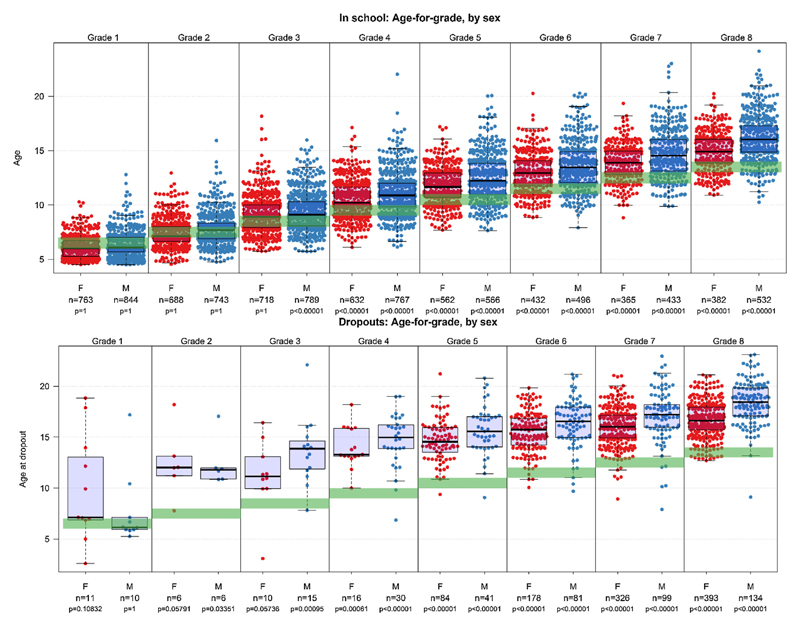
Age-for-grade heterogeneity among those in and out-of-school in 2010, by sex. The age-for-grade distributions for students in and out of primary school in 2010. Those in school in 2010 are shown in the upper panel. The age at dropout and highest grade attained among those who dropped out of primary school in the past 10 years (2000–2010), are shown in the lower panel. Females and males are shown separately. Each dot represents a student and the box plots show the median, interquartile range and outer limits of age distributions within each grade. The horizontal bar shows the official age-for-grade. The average age-for-grade increases by grade for both males and females with increasing grade and is higher among those who drop out than those who remain in school. The Wald test p-values is for the comparison of the median age-for-grade with the official age-for-grade.

The age-for-grade distribution is shown in Fig. 2. The top figure shows the distribution of age-for-grade among those in primary school, and the lower figure shows the distribution for those out of school in 2010. Among those in school there is a greater heterogeneity of ages at higher grade levels, with a significant deviation from the official age-for-grade (the horizontal band highlighted in green), from grade 3 onwards (p < 0.01 Wilcoxon rank sum test). For example, inter-quartile ranges for boys in grade 1 ranged between 4 and 9 years, as compared to the distribution for boys in grade 8 which ranged between 11 and 21 years. There are higher proportions of under-age students in the early stages and higher proportions of over-age students, especially boys, at later stages. The deviation of the median age from the offificial age-for-grade increases with each subsequent grade, ranging from about 1 year underage in grade 1 to about 2–3 years overage in grade 8. Age-for-grade for those previously in school was calculated based on the age (or year) at leaving school and the highest grade attended and was restricted to those who reported leaving school within the last 10 years (2000–2010) to limit recall bias. Those who dropped out over the last ten years were mostly in grades 5–8 and were 2–5 years over-age for their grade in all grades (p < 0.01). The median age at dropout among those out of school was 16 for girls and 17 for boys.

Subsequent analyses concentrated on those respondents who were still in school the following year (as the outcome was repetition in the following year). A further 272 respondents were omitted due to missing school-level data. The remaining 8174 participants were enrolled in 24 primary schools in 2010 (Fig. 1). This includes those who had completed grade 8 and were out of school the following year, who were categorised as non-repeaters (n = 8), as they had performed similarly to those who progressed to secondary.

Table 2 shows their characteristics. 54% were male and nearly half were aged under 10 and in grades 1–3. While 47% were at the official age-for-grade, 12% were underage and 41% were overage. The majority of the students (Table 3) were in schools that were considered high performing (63% in schools with >75% pass rate at the grade 8 terminal exam); complete (71%); and funded by church groups (86%). More than half of the schools had fewer than 25% female teachers on staff (n = 14 or 53% students) and student-teacher ratios <60:1 (n = 15 or 53% students). Seventeen schools (84% students) had access to water (piped/borehole) but only two schools (16% students) had any electricity.

### 3.2 Grade repetition

Overall, 39% of participants reported repeating their current grade when interviewed the following year, with little variation between boys and girls. Nearly half the students in grades 1–3 (41%) had repeated their grade at least once (Fig. 2), with almost one-third of students in grade 1 having repeated twice or more. Almost half of grade 8 students had repeated at least once with 72 students (10%) having repeated twice or more. The proportion of repeaters was highest among students in schools which were smaller, incomplete, with higher student-teacher ratios, no female teachers and with low school performance (Table 3). Schools that had access to water and electricity had lower proportions of repeaters.

**Fig. 3:**
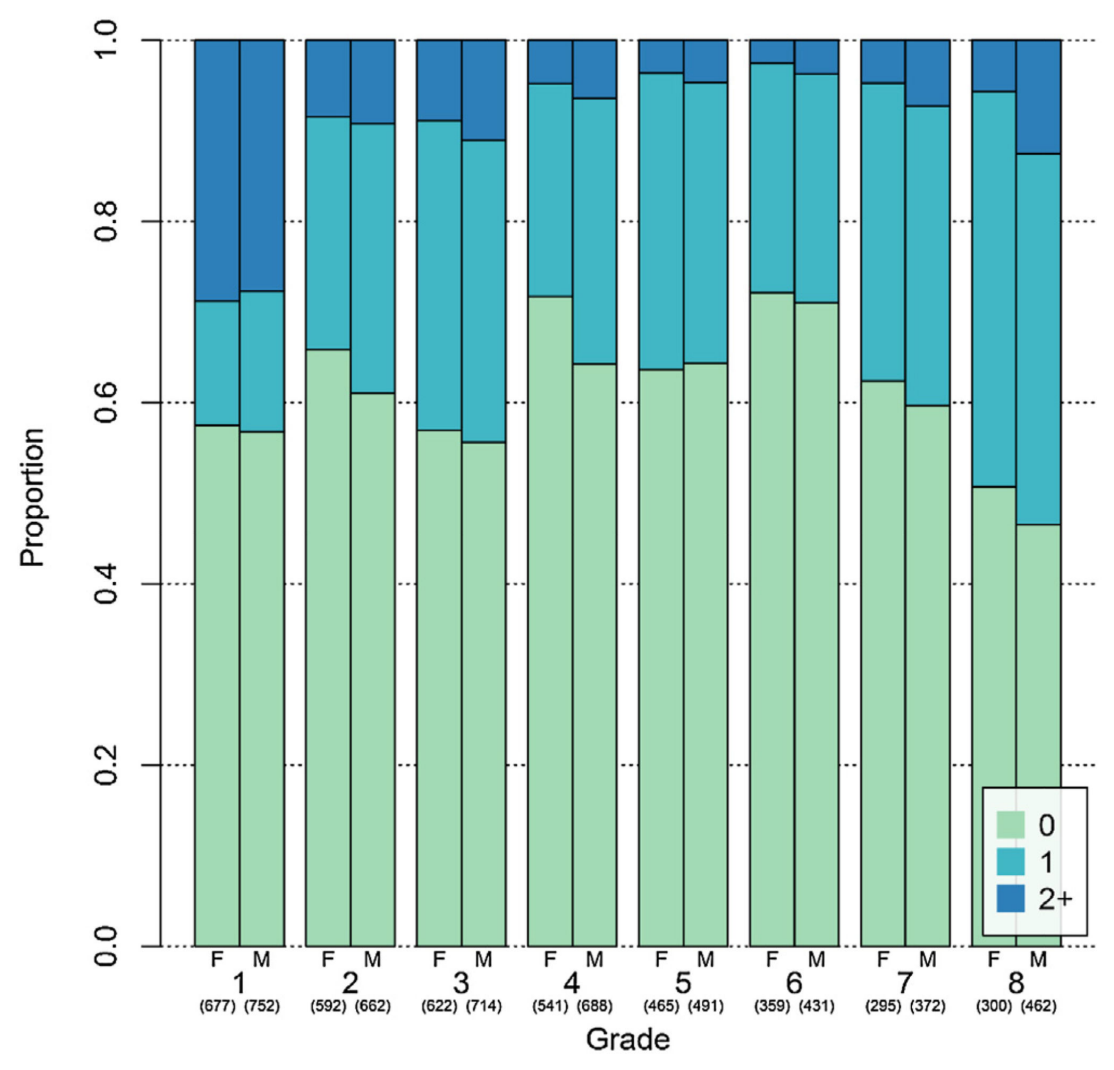
Grade Repetition, by grade and sex. Fig. 3 shows the proportions of repeaters and the extent of repetition, by grade and sex. Repetition is highest in early (grades 1–3) and later stages (grades 7–8), with almost 30% of grade 1 students repeating their current grade two or more times.

**Fig. 4:**
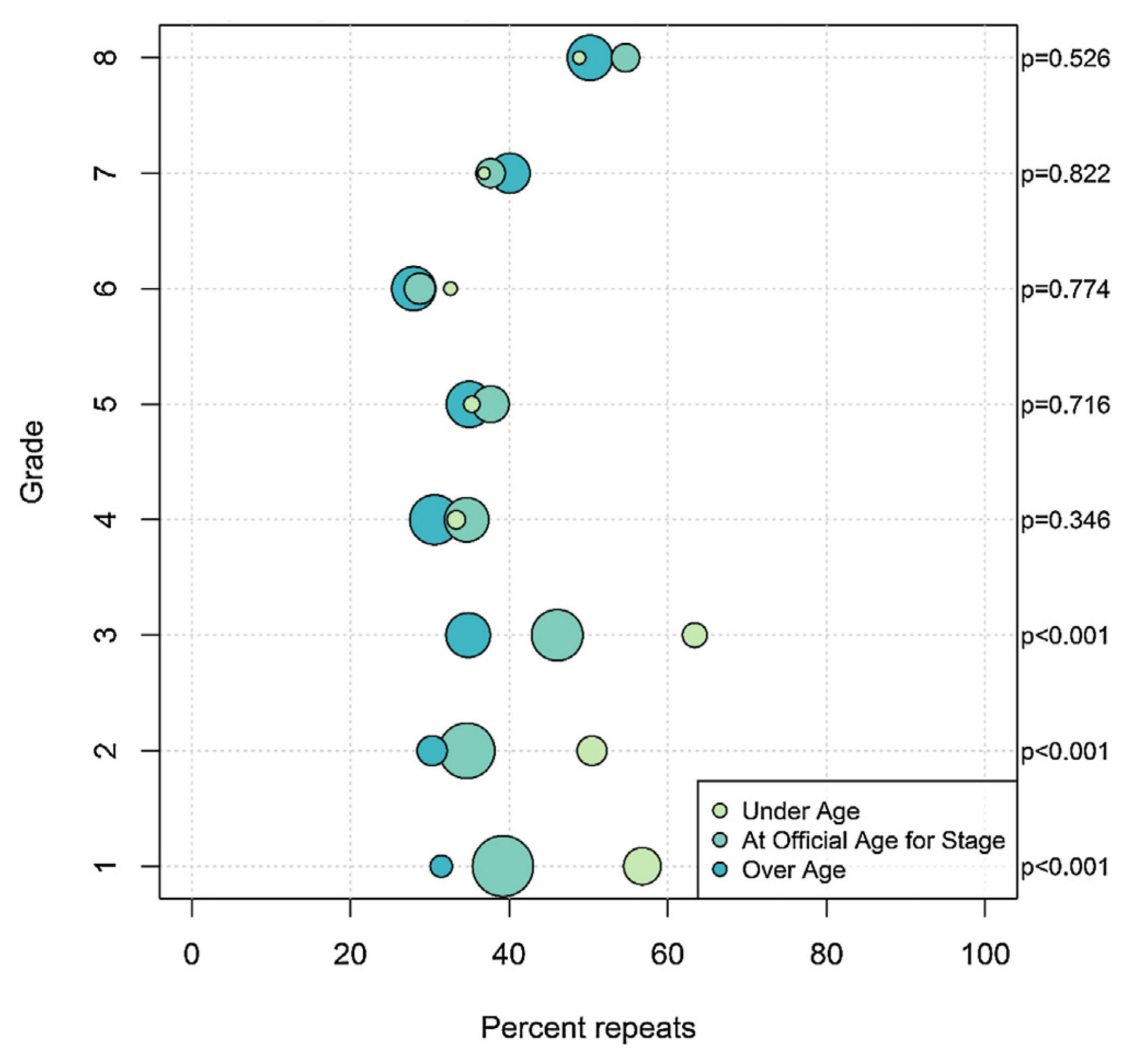
Repetition by age-for-grade and grade. Fig. 4 shows the proportion repeating in each grade in 2011 by age-for-grade in 2010. The size of the circle is proportional to the number of students within that group. The p-values (Wald test) are for the comparison of the risk of repetition by age-for-grade within each grade.

Table 4 examines the risk factors for grade repetition in the following year, by gender showing both the univariable and multivariable analyses. Most associations were similar in the crude analysis and after adjustment for other factors. Grade repetition is more common among those under-age for their grade; and at early and later stages (grades 1–3, grades 7–8) of school. It was less common among those over-age by two or more years for their grade. Living without their mother and in lower quality dwellings increased the risk of repetition. Those whose fathers had completed at least primary education were less likely to repeat. These trends persisted after adjusting for other individual, household and school-level factors (Figs. 3 and 4).

**For girls**, repetition was more common among those who were absent for at least a week within the last four weeks of school. Living without their father; or with one or more children (<6years) within the same household increased their risk of repetition. Girls who studied in schools with a higher female teacher ratio (>50%); or with access to water were less likely to repeat. **For boys**, repetition was less common among those who were overage by at least one year; or living in a higher quality dwelling. The risk of repetition was higher among those who studied in schools with pupil: teacher ratio of >60:1; and was higher in high or low performing schools compared to mid performing schools.

### 3.3 Age-for-Grade heterogeneity and grade repetition

The association between age-for-grade and repetition varied by school grade as shown in Fig. 4. Grade repetition is highest in early (grades 1–3: 41%) and later (grades 7–8: 46%) stages of schooling; and lowest in grades 4–6 (33%). Almost 60% of under-age students in grade 1 (Fig. 4) repeated their current grade, with higher proportions of under-age repetitions at early stages (p < 0.01). Table 5a,b examine the association of age-for-grade heterogeneity and grade repetition, stratified by early and later (grades 4–8) stages of schooling. The majority of the risk factors for grade repetition noted above were observed at early but not later stages of schooling.

#### 3.3.1 At early stages (Grades 1–3)

Those under-age for their grade at early stages were twice as likely to repeat a grade (girls: adj OR 2.01 p < 0.01; boys: adj OR 2.25 p < 0.01) as those at the official age-for-grade. Being over-age at early stages was associated with lower repetition (girls: adj OR 0.63 p < 0.01; boys: adj OR 0.67 p < 0.01) for both boys and girls. Repetition was less common among those whose fathers have completed at least primary. Those living without the mother or with one or more children below the age of six, within the same household, or in worse housing were at greater risk of repeating their current grade. School-level factors, like high student-teacher ratios (>60:1); more female teachers (for boys only); and access to water (girls only) were associated with higher repetition at early stages. School absenteeism showed no association with age-for-grade and grade repetition at early stages, even after adjusting for other risk factors. Repetition was lowest in mid-ranking schools, for boys.

#### 3.3.2 At later stages (Grades 4–8)

Fewer risk factors were identified that contribute to grade repetition at later stages. There was no evidence of effect for age-for-grade heterogeneity on grade repetition at later stages, after adjusting for other risk factors, for either boys or girls. Father’s education reduced the risk of repetition for both boys and girls. ***For girls at later stages***, being absent for a week or more; or living with either parent, increased their risk of repetition. Repetition was less common among girls studying in a high-performing school and in schools with greater proportions of female teachers (>50%). ***For boys only, at later stages:*** repetition was more common for those living in a poor quality dwelling; and studying in schools with higher pupil-teacher ratios >60:1.

## 4 Discussion

A large proportion of school children repeat grades leading to substantial age heterogeneity in each school grade. However we found no evidence that being over-age in itself leads to repetition. Being under-age at early stages (grades 1–3) is a significant risk factor for grade repetition among boys and girls; whereas those over-age at early stages progress more quickly. The outperformance of over-age children over under-age children is probably because over-age children are motivated to perform better at early stages; are more familiar with the material and actually perform better; or are automatically advanced to the next grade by teachers in the hope that they catch up at later grades. At later stages (grades 4–8), there is no association between age-for-grade heterogeneity and grade repetition, although being over-age by 2+ years is associated with dropping out.

Our findings concur with a previous descriptive study using DHS data, which showed that countries with low overall promotion rates, like Malawi, had higher over-age progressions at early stages; while countries with higher promotion rates showed less distinct patterns of promotion by relative age (Wils et al., 2009). In the Malawi DHS, as in our data, under-age children had higher repetition rates in the early grades, especially grade 1. Being over-age was not a risk factor for repetition, but was associated with dropping out of school. In the DHS data analysis, the effect of age-for-grade heterogeneity on grade repetition was not adjusted for other co-variates. This contrasts with earlier findings from Mozambique which showed that being over-age at school entry is a risk factor for grade repetition (Nonoyama-Tarumi et al., 2010; Wils, 2004). This is not to deny that over-age enrolment into school has an adverse effect on schooling, but that being under-age or over-age in school has different implications on grade repetition, depending on the context of schooling (UNESCO, 2012).

Slow progression or disinterest in school at early stages may have a cumulative effect on schooling at later stages. Repetition in grades 7–8 is high although there is no detectable effect of being under-age/over-age as almost two-thirds of students are over-age at this stage. Repetition maybe high on account of “voluntary” repetitions by students who choose to repeat their grade in order to improve their performance in the terminal year exam, or due to unaffordability of exam fees (Ndaruhutse et al., 2008; Wils et al., 2009). Delays in progression at later grades leads to wider age heterogeneity in the class, which may have a “peer-effect” on performance. In South Africa, Lam et al. showed that interacting with older peers in class had an adverse influence on in-school pregnancy (Marteleto et al., 2008). Age at enrolment contributes to age-for-grade heterogeneity at early and later stages. Under-age school entry may take place to off-set the lack of adequate pre-school facilities; to ease the provision of child-care by older siblings who attend school or to provide children an early exposure to the school setting (Fentiman et al., 1999b). Late school entries take place on account of parental perceptions of the child’s readiness for school (physical, social, cognitive), financial need; and distance to school (Nonoyama-Tarumi et al., 2010).

Socio-economic and school-level factors that were associated with age-for-grade heterogeneity and grade repetition were not gendered but varied by stage of schooling. The links between socioeconomic status of households, school quality and schooling have been well studied (Gomes-Neto, 1991; Glick and Sahn, 2000, 2010; Patrinos and Psacharopoulos, 1992; Sackey, 2007), although less so in understanding the risk factors for grade repetition by stage of schooling. Educated fathers or living with both parents may provide a more enabling environment at home which is supportive of schooling and foster learning, especially at early stages (Booth, 1996; Glick and Sahn, 2000). Living with either parent (only father/only mother) may be a risk factor as the absence of the mother may imply greater domestic responsibilities that may conflict with schooling; and the absence of the father may imply greater financial burden on the household. This is consistent with our finding that children at early stages of school, who live with at least one child below the age of six, may experience more child-rearing and domestic duties which reduces time from school, leading to poor performance and higher repetitions in school. As the age range of those in early stages is quite wide (between 5 and 12 years) this may reflect the allocation of household duties to younger members of the household, while income-earning responsibilities are more likely to be allocated to those at older age groups (Fentiman et al., 1999b). The effect of living in a poorer quality dwelling, especially among boys at later stages, concurs with previous evidence on household economic status and school participation, especially among students who volunteer to repeat a grade due to lack of exam fees. Dwelling score is a crude measure of relative socio-economic status so we would expect a stronger correlation with more detailed measures. Despite efforts to universalise primary education, households still incur higher direct (exam fees, textbooks, transportation) and indirect costs of schooling at later stages.

Schools which have lower student-teacher ratios (<60:1), higher female teacher ratios (>50%) ratios, improved infrastructure (access to water) may reduce the risk of repetition, especially for girls at early stages. Access to water in schools and links to menstrual management may enable attendance and participation in schools for girls at later stages, though there is no clear evidence of this association (Birdthistle et al., 2011). Positive links with access to water in school and grade repetition at early stages may be related to other factors of school-quality, like proximity to roads/businesses, which may attract more qualified teachers or improve teacher attendance. School performance showed a non-linear association with repetition, especially for boys at early stages, which was high irrespective of whether they were enrolled in low or high performing schools. While the higher risk of repetition at lower performing schools is understandable, the higher risk of repetition at high performing schools may be on account of the schools’ need to maintain a higher level of performance at all stages of schooling, by raising performance thresholds and compelling students to repeat.

Early academic performance is an important determinant of performance at later grades(Glick and Sahn, 2010). Higher risk of repetition among under-age students and the progression of over-age students in early grades, leads to a growing pool of over-age students at later stages who are approaching the age of adolescence. For example, the median age of students in grade 6 is around 13.5, compared to the official age-for-grade of 11–12 years. Previous studies in northern Karonga have shown that girls who reach menarche before the age of 14 are more likely to have sex, get pregnant and marry sooner; and are less likely to complete school than those who reach menarche at older ages (Glynn et al., 2010). While being overage at later stages is not a significant risk factor for repetition at later stages, academic failure when overage may lead to dropping out of school as a preferred choice over repetition. Given the low number of dropouts observed in 2011 (n = 178, 2%), which may be indicative of the dynamic nature of dropping out of school (Hunt, 2008), questions around the effect of age-for-grade heterogeneity on dropout and other competing risk factors (like first sex, pregnancy, marriage) are better explored using longitudinal data.

One of the main limitations of this study is the use of self-reported data on grade repetition, which may be prone to social desirability bias or measurement error. However, given the longitudinal nature of the KPS data, any inconsistencies or missing data were corrected using current/previous years’ schooling status and repetition data, thereby minimising bias. There may also be concerns around the accuracy of age as reported by respondents, which was reported at the baseline census and only asked for those newly migrating into the DSS catchment area. Those respondents who could not provide a precise date of birth, mostly older generations, had the dates and month of birth centred for the middle of the month/year. Being part of a larger demographic surveillance site which has been collecting data on births, deaths and migrations since 2002, registration of vital events remains important and hence more accurate for the younger age-groups, who are the target group for this study. In the absence of standardized tests in schools, our study uses grade repetition as a proxy for school performance. This raises concerns about the measures of performance used in schools and how accurately a teacher’s judgement on whether a student should repeat a grade or not is a true reflection of the student’s ability or competency for that grade (Ndaruhutse et al., 2008). Other school-level risk factors for repetition which were not measured in this study, like instructional time, teacher attendance, teachers’ motivation and self-efficacy, need to be considered for future studies.

This study further raises the issue of grade repetition as a practice, and whether it is a necessary and a sufficient condition to improve student performance. Repetition is considered beneficial in attaining homogeneity of ability within a classroom which is easier for teachers to manage (Fentiman et al., 1999a) although this assumes that the methods of choosing who should repeat are reliable which may not be the case (Bernard et al., 2005). Those in favour of repetition claim that it provides flexibility for “slow learners” or students who need more time to master the course content. It provides flexibility for students to meet their individual learning needs, especially in schools where the language of instruction differs from their mother tongue (UNESCO, 1998). But repetition prolongs the duration of schooling, which conflicts with the period of adolescence(Glynn et al., 2010); and can affect self-esteem and motivation of students to persist (Ndaruhutse et al., 2008). The economic argument against grade repetition has revolved around increased school inefficiencies and the higher costs associated with lower/delayed entryofgraduates intothe work force, to contribute to the productivity of the economy. In 2004, a World Bank study assessed that a 1% reduction in repetition in Malawi would result in an annual saving of around MK 30 million (around $300,000) (World Bank, 2004). The opportunity cost of staying in school also increases with age (Fentiman et al., 1999b), especially in agrarian or subsistence economies where families perceive a higher return on adolescent’s labour by working on the farm or in the household, rather than attending school.

While automatic or social promotion in school is gaining approval and implemented in several countries (like Mauritius, Seychelles, Zimbabwe), its relevance within a developing country context is still questionable, given the growing paucity of qualified teachers, teaching materials and resources for existing learners, and most importantly remedial teaching needs for those retained. While neither grade repetition nor automatic promotion have shown an impact on student performance (Ndaruhutse et al., 2008; UNESCO, 2012), repetitions will have fewer children progressing through school with higher levels of dropout at later stages; while automatic/social promotion will result in more students progressing through school, but perhaps with a minimum level of learning/mastery achieved upon completion. In Malawi, efforts to introduce less stringent, yet uniform conditions for promotion (50% pass rate in 2 subjects) between certain grades (grades 4, 6, 8) are being deliberated upon, though not yet implemented (World Bank, 2010, p. 60). The recently introduced Education Sector Implementation Plan-II (2013–2018) suggests a 10% cap on repetition in primary schools (Ravishankar et al., 2015), though the effects on performance are yet to be understood. Irrespective of whether country policies adopt a practice of grade repetition or automatic promotion, the focus needs to be directed towards school quality and meeting the diverse (age, sex, cognitive) learning needs of students to enable timely progression through school.

## 5 Conclusion

Timely progression through school is important, not only for school performance and completion, but also for ensuring that reasonable educational levels are reached before the onset of other life transitions for adolescents. Although we did not find that being over age was a risk factor for repetition, it results from repetition and leads to increased drop out. Most students are not dropping out at young ages, but they are dropping out undereducated. The levels of repetition were extremely high at all stages, implying poor learning. Many risk factors were similar for boys and girls. From a policy perspective, it is critical to address the varied learning needs of children, through greater investments in managing multi-age teaching, quality resource allocations (timely provision of textbooks, infrastructure and qualified teachers), remedial learning and focus on reading, writing and numeracy skills at early stages that equip students to progress through each grade on time.

## Figures and Tables

**Table 1:** Characteristics of respondents in 2010, by schooling status in 2011.

Characteristics	In School		Dropout		Departed/Died		Missing		Total
Age	n	%	n	%	n	%	n	%	n
5–9	3801	88.6	25	0.6	301	7.0	161	3.8	4288
10–14	3576	88.0	41	1.0	321	7.9	126	3.1	4064
15–24	1070	78.7	112	8.2	120	8.8	58	4.3	1360
Grade									
P1-3	4031	88.7	39	0.9	304	6.7	171	3.8	4545
P4-6	2979	86.2	72	2.1	282	8.2	122	3.5	3455
P7-8	1437	83.9	67	3.9	156	9.1	52	3.0	1712
Sex									
Female	3867	85.1	58	1.3	427	9.4	190	4.2	4542
Male	4580	88.6	120	2.3	315	6.1	155	3.0	5170
Age-for-Grade									
Under Age	1016	88.0	9	0.8	92	8.0	37	3.2	1154
At Official Age	3951	88.8	18	0.4	327	7.3	154	3.5	4450
Over Age-1 yr	1438	89.1	14	0.9	113	7.0	49	3.0	1614
Over Age-2 + yr	2042	81.9	137	5.5	210	8.4	105	4.2	2494
TOTAL	8447		178		742		345		9712

**Table 2:** Characteristics of study population between ages 5–24yrs currently in school.

Characteristics	n	%
All	8174	
Sex		
Female	3733	45.7
Male	4441	54.3
Age-for-Grade		
Under Age	976	11.9
At Official Age	3823	46.8
Over Age-1 yr	1395	17.1
Over Age-2 + yr	1980	24.2
Age		
5–9	3675	45.0
10–14	3470	42.5
15–24	1029	12.6
Highest grade attended		
*Early stages* P1-3	3897	47.7
P 4-6	2914	35.6
*Later stages* P7-8	1363	16.7
Age at Enrolment		
On time (6yrs)	5356	65.5
Under Age (<6yrs)	1766	21.6
Over Age (>6 yrs)	1048	12.8
Dwelling score		
1 (Worst)	3882	47.5
2	2142	26.2
3 (Best)	2150	26.3

**Table 3:** Descriptive characteristics of 24 primary schools in Karonga district, northern Malawi.

School Characteristics		No. of schools n (%)	% of students	Repetition: No. repeating/no. of students (%)
	No. of primary schools	24		
	No. of students enrolled	8174		
School size (no. of students)				
	*< = 500*	10(42)	24	820/1934(42)
	*500–1000*	12(50)	58	1838/4762(39)
	>1000	2(8)	18	531/1478(36)
Student-Teacher Ratio				
	*< = 60*	15(62)	53	1590/4212(38)
	*60–80*	4(17)	30	568/1476(39)
	*>80*	5(21)	18	1031/2486(42)
% Female teachers				
	*None*	2(8)	3	113/252(45)
	*< 25%*	12(50)	50	1648/4042(41)
	*25–50%*	6(25)	30	915/2410(38)
	*>50%*	4(17)	18	513/1470(35)
School Rank^a^				
	*Low (*<*50%)*	7(29)	11	450/895(50)
	*50–75%*	5(21)	26	717/2125(34)
	*High (*>75%*)*	12(50)	63	2022/5154(39)
Funding source				
	*Government*	4(17)	12	375/994(38)
	*Religious Authority*^b^	19(79)	86	2760/7037(39)
	*Private*	1(4)	2	54/143(38)
Complete schools^c^				
	*No*	7(29)	11	450/895(50)
	*Yes*	17(71)	89	2739/7279(38)
Access to . . .				
*Water*	*No*	7(29)	16	617/1326(47)
	*Yes(piped/borehole)*	17(71)	84	2572/6848(38)
Electricity	*No*	22(92)	84	2724/6850(40)
	*Yes*	2(8)	16	465/1324(35)

a School rank is based on the proportion of students who pass the Primary School Leaving Examination (PSLE) in Grade 8.

b Religious Authority schools include those funded by Roman Catholic (RC), Church of Central Africa Presbyterian (CCAP), Anglican, Seventh Day Adventist churches.

c Complete schools are schools that provide all eight grades of primary.

**Table 4:** Risk factors for grade repetition in the following year, for 8174 primary school students, by sex.

Variables		Females (N = 3733)								Males (N = 4441)							
		n/N	%	Crude OR	CI	p*	Adj OR¥	p	CI	n/N	%	Crude OR	CI	p*	Adj OR¥	p	CI
All		1402/3733	37.6							1787/4441	40.2						
Grade	P1-3	737/1834	40.2	1.44	1.23–1.67	<0.01	1.18	0.06	0.99–1.39	878/2063	42.6	1.42	1.24–1.63	<0.01	1.22	0.01	1.05–1.43
	P4-6	415/1331	31.2	1			1			535/1583	33.8	1			1		
	P7-8	250/568	44.0	1.81	1.47–2.22		1.94	0.00	1.56–2.40	374/795	47.0	1.72	1.44–2.05		1.94	0.00	1.61–2.33
Age-for-Grade																	
	Under Age	262/538	48.7	1.66	1.36–2.02	<0.01	1.76	0.00	1.43–2.17	235/438	53.7	1.76	1.42–2.18	<0.01	1.93	0.00	1.55–2.41
	At Ofﬁcial Age	709/1897	37.4	1			1			776/1926	40.3	1			1		
	Over Age-1 + yr	215/615	35.0	0.88	0.73–1.07		0.86	0.15	0.70–1.06	281/780	36.0	0.83	0.70–0.99		0.79	0.01	0.66–0.95
	Over Age-2 +yr	216/683	31.6	0.76	0.63–0.92		0.71	0.00	0.58–0.88	495/1297	38.2	0.9	0.77–1.04		0.81	0.01	0.68–0.96
Absenteeism																	
	0 wk	1136/3090	36.8	1		0.01	1			1473/3656	40.3	1		0.96	1		
	1 + wk	252/596	42.3	1.27	1.06–1.52		1.24	0.02	1.03–1.50	296/733	40.4	1	0.85–1.18		1.04	0.63	0.88–1.23
	Missing	14/47	29.8	0.78	0.41–1.47					18/49	36.7	0.85	0.47–1.53				
Dwelling score																	
	1 (Worst)	707/1761	40.1	1.17	0.99–1.38	0.05	1.21	0.04	1.01–1.44	921/2121	43.4	1.18	1.02–1.37	<0.01	1.21	0.02	1.04–1.41
	2	352/966	36.4	1			1			464/1176	39.5	1			1		
	3 (Best)	343/1006	34.1	0.97	0.80–1.17		0.97	0.78	0.79–1.19	402/1144	35.1	0.83	0.70–0.99		0.79	0.01	0.66–0.94
Living w/																		
	Both parents	859/2257	38.1	1		0.02	1			1122/2730	41.1	1		0.19	1		
	Father only	87/189	46.0	1.43	1.05–1.93		1.64	0.00	1.20–2.24	131/294	44.6	1.19	0.93–1.52		1.30	0.04	1.01–1.67
	Mother only	224/598	37.5	1.01	0.84–1.22		1.26	0.04	1.01–1.56	252/657	38.4	0.93	0.78–1.11		1.02	0.88	0.83–1.25
	Neither parent	232/689	33.7	0.85	0.71–1.02		1.26	0.04	1.02–1.57	282/760	37.1	0.89	0.75–1.06		1.22	0.05	1.00–1.49
Children <6yrs in hh																	
	0	344/1053	32.7	1		<0.01	1			516/1368	37.7	1		0.03	1		
	1+	1058/2680	39.5	1.34	1.15–1.56		1.28	0.00	1.08–1.51	1271/3073	41.4	1.16	1.01–1.32		1.1	0.21	0.95–1.27
Father’s Ed																	
	None/ < Grade 8	697/1652	42.2	1		<0.01	1			899/2067	43.5	1		<0.01	1		
	At least Primary	666/1987	33.5	0.72	0.62–0.83		0.65	0.00	0.55–0.76	843/2257	37.4	0.80	0.71–0.91		0.75	0.00	0.64–0.87
	Missing	39/94	41.5	1.03	0.67–1.58					45/117	38.5	0.85	0.58–1.26				
Pupil-Teacher Ratio																	
	<60:1	730/1913	38.2	1		0.7	1			860/2299	37.4	1		0.22	1		
	60–80:1	225/661	34.0	0.83	0.51–1.34		1.26	0.15	0.92–1.73	343/815	42.1	1.37	0.89–2.10		1.56	0.00	1.16–2.11
	>80:1	447/1159	38.6	1.02	0.68–1.55		1.13	0.40	0.85–1.50	584/1327	44.0	1.29	0.88–1.88		1.27	0.09	0.96–1.68
Female Teacher																		
	<25%	784/1965	39.9	1		0.04	1			977/2329	41.9	1		0.46	1		
	25–50%	417/1097	38.0	0.94	0.65–1.36		1.32	0.07	0.98–1.78	498/1313	37.9	0.8	0.54–1.17		1	0.99	0.74–1.34
	>50%	201/671	30.0	0.57	0.37–0.88		0.70	0.03	0.51–0.97	312/799	39.0	0.85	0.54–1.33		1.26	0.15	0.92–1.72
School Rank																	
	Low < 50%	216/411	52.6	2.37	1.67–3.37	<0.01	1.33	0.26	0.81–2.19	234/484	48.3	1.78	1.20–2.64	0.01	2.02	0.00	1.27–3.22
	Mid < 75%	309/970	31.9	1			1			408/1155	35.3	1			1		
	High:>75%	877/2352	37.3	1.31	0.99–1.74		1.04	0.81	0.74–1.47	1145/2802	40.9	1.45	1.04–2.03		1.59	0.01	1.14–2.23
Access to water																	
	No	284/593	47.9	1		<0.01	1			333/733	45.4	1		0.26	1		
	Yes(piped/borehole)	1118/3140	35.6	0.60	0.43–0.82		0.56	0.00	0.38–0.83	1454/3708	39.2	0.81	0.57–1.16		0.92	0.67	0.64–1.33
Mother’s Ed																	
	None/<Primary	978/2532	38.6	1		0.08				1268/3057	41.5	1		0.09			
	At least Primary	405/1165	34.8	0.88	0.76–1.02					507/1343	37.8	0.9	0.78–1.03				
	Missing	19/36	52.8	1.79	0.92–3.48					12/41	29.3	0.61	0.31–1.20				
Distance to school																	
	< = 1km	807/2187	36.9	1		0.5				1027/2600	39.5	1		0.2			
	> 1km	582/1514	38.4	1.05	0.91–1.22					740/1801	41.1	1.09	0.96–1.25				
	Missing	13/32	40.6	1.04	0.42–2.60					20/40	50.0	1.53	0.67–3.49				
Access to Electricity																	
	No	1190/3131	38.0	1		0.48				1534/3719	41.2	1		0.19			
	Yes	212/602	35.2	0.81	0.45–1.45					253/722	35.0	0.7	0.41–1.19				

* Summary p-values.MissingobservationsomittedfromWaldtest.

¥ Adjustedforindividual,householdandschool-levelfactors.

**Table 5:** Association between age-for-grade heterogeneity and grade repetition for 8,174primary school students, by stage and sex.

a.		Females (N = 3733)													
		Early Stages (grades 1–3) n = 1834							Later Stages (grades 4–8) n = 1899						
Variables		n/N	%	crude OR	CI	adj OR¥	p	CI	n/N	%	crude OR	CI	adj OR¥	p	CI

All		737/1834	40.2						665/1899	35.0					
Age-for-Grade	Under Age	212/392	54.1	2.03	1.59–2.58	2.01	0.00	1.56–2.59	50/146	34.2	0.96	0.65–1.40	1.08	0.69	0.73–1.61
	At Official Age	426/1121	38.0	1		1			283/776	36.5	1		1		
	Over Age-1+ yr	99/321	30.8	0.67	0.51–0.89	0.63	0.00	0.47–0.84	332/977	34.0	0.88	0.72–1.07	0.87	0.20	0.70–1.08
Absenteeism	Not absent	592/1491	39.7	1		1			544/15997	34.0	1		1		
	1+ wk	138/317	43.5	1.15	0.90–1.48	1.16	0.26	0.89–1.51	114/279	40.9	1.39	1.07–1.82	1.39	0.02	1.05–1.83
Dwelling score	1 (Worst)	402/955	42.1	1.26	1.00–1.60	1.26	0.07	0.98–1.60	305/806	37.8	1.07	0.84–1.37	1.07	0.62	0.83–1.37
	2	177/475	37.3	1		1			175/491	35.6	1		1		
	3 (Best)	158/404	39.1	1.19	0.90–1.58	1.19	0.25	0.89–1.61	185/602	30.7	0.82	0.63–1.07	0.90	0.44	0.68–1.18
Living w/	Both parents	492/1204	40.9	1		1			367/1053	34.9	1		1		
	Father only	46/95	48.4	1.39	0.91–2.12	1.73	0.02	1.11–2.69	41/94	43.6	1.50	0.97–2.33	1.56	0.05	1.00–2.42
	Mother only	100/278	36.0	0.84	0.64–1.11	1.12	0.46	0.82–1.53	124/320	38.8	1.21	0.93–1.57	1.41	0.03	1.03–1.94
	Neither parent	99/257	38.5	0.93	0.70–1.24	1.70	0.00	1.21–2.39	133/432	30.8	0.84	0.65–1.07	1.04	0.79	0.78–1.39
Children <6yrs in hh	0	113/367	30.8	1		1			231/686	33.7	1		1		
	1+	624/1467	42.5	1.67	1.30–2.15	1.66	0.00	1.26–2.18	434/1213	35.8	1.11	0.91–1.35	1.04	0.71	0.84–1.29
Father’s Ed	None/ < Primary	395/882	44.8	1		1			302/770	39.2	1		1		
	At least Primary	333/923	36.1	0.73	0.60–0.88	0.63	0.00	0.50–0.79	333/1067	31.2	0.71	0.58–0.86	0.67	0.00	0.53–0.86
Pupil-Teacher Ratio	<60:1	368/934	39.4	1		1			362/979	37.0	1		1		
	60–80:1	115/292	39.4	0.99	0.57–1.70	1.59	0.01	1.11–2.27	110/369	29.8	0.77	0.45–1.32	1.04	0.89	0.61–1.78
	>'80:1	254/608	41.8	1.19	0.76–1.88	1.45	0.02	1.06–2.00	193/551	35.0	0.92	0.57–1.47	0.80	0.39	0.49–1.32
Female Teacher(%)	<25%	422/1004	42.0	1		1			362/9610	37.7	1		1		
	25–50%	224/554	40.4	0.95	0.62–1.44	1.61	0.00	1.16–2.25	193/543	35.5	0.87	0.56–1.36	0.94	0.83	0.55–1.61
	>50%	91/276	33.0	0.59	0.36–0.99	0.79	0.23	0.53–1.16	110/395	27.8	0.58	0.35–0.97	0.61	0.08	0.35–1.07
School Rank	Low < 50%	142/250	56.8	2.52	1.80–3.51	1.48	0.18	0.83–2.64	74/161	46.0	1.90	1.08–3.34	1.15	0.75	0.49–2.70
	Mid 50–75%	148/431	34.3	1		1			161/539	29.9	1		1		
	High:>75%	447/1153	38.8	1.21	0.95–1.56	0.90	0.61	0.61–1.34	430/1199	35.9	1.37	0.89–2.12	1.31	0.36	0.73–2.34
Access to water	No	158/310	51.0	1		1			126/283	44.5	1		1		
	Yes (piped/borehole)	579/1524	38.0	0.59	0.41–0.84	0.55	0.01	0.34–0.86	539/1616	33.4	0.65	0.42–1.00	0.65	0.20	0.33–1.26

b.		Males (N = 4441)													
		Early Stages (grades 1–3) n = 2063							Later Stages (grades 4–8) n = 2378						
Variables		n/N	%	crude OR	CI	adj OR¥	p	CI	n/N	%	crude OR	CI	adj OR¥	p	CI

All		878/2063	42.6						909/2378	38.2					
Age for Grade	Under Age	187/317	59.0	2.12	1.64–2.75	2.25	0.00	1.71–2.95	48/121	39.7	1.09	0.73–1.63	1.23	0.33	0.81–1.86
	At Official Age	513/1236	41.5	1		1			263/690	38.1	1		1		
	Over Age-1+ yr	178/510	34.9	0.72	0.57–0.90	0.67	0.00	0.53–0.84	598/1567	38.2	0.99	0.82–1.19	0.96	0.65	0.78–1.16
Absenteeism	Not absent	718/1686	42.6	1		1			755/1972	38.3	1		1		
	1+ wk	154/359	42.9	0.99	0.78–1.25	1.00	1.00	0.78–1.28	142/375	37.9	1.02	0.81–1.29	1.02	0.89	0.80–1.29
Dwelling score	1 (Worst)	471/1083	43.5	0.92	0.74–1.15	0.95	0.64	0.76–1.19	450/1038	43.4	1.44	1.17–1.78	1.47	0.00	1.19–1.83
	2	242/519	46.6	1		1			222/657	33.8	1		1		
	3 (Best)	165/461	35.8	0.64	0.49–0.84	0.59	0.00	0.45–0.78	237/683	34.7	1.04	0.82–1.31	1.02	0.84	0.81–1.30
Living w/	Both parents	606/1380	43.9	1		1			516/1350	38.2	1		1		
	Father only	61/117	52.1	1.41	0.96–2.08	1.59	0.02	1.07–2.37	70/177	39.5	1.11	0.81–1.54	1.09	0.59	0.79–1.52
	Mother only	107/291	36.8	0.78	0.60–1.02	0.97	0.84	0.72–1.31	145/366	39.6	1.11	0.87–1.41	1.07	0.66	0.80–1.42
	Neither parent	104/275	37.8	0.79	0.61–1.04	1.29	0.11	0.94–1.77	178/485	36.7	1.02	0.81–1.27	1.13	0.36	0.87–1.47
Children<6yrs in hh	0	156/443	35.2	1		1			360/925	38.9	1		1		
	1+	722/1620	44.6	1.45	1.16–1.81	1.30	0.03	1.02–1.66	549/1453	37.8	0.96	0.81–1.14	0.94	0.54	0.79–1.13
Father’s Ed	None/ < Primary	470/1010	46.5	1		1			429/1057	40.6	1		1		
	At least Primary	397/1026	38.7	0.74	0.61–0.89	0.70	0.00	0.56–0.86	447/1234	36.2	0.87	0.73–1.04	0.82	0.06	0.66–1.01
Pupil-Teacher Ratio	<60:1	421/1073	39.2	1		1			439/1226	35.8	1		1		
	60–80:1	139/354	39.3	0.96	0.62–1.49	1.23	0.31	0.82–1.85	204/461	44.3	1.63	1.03–2.59	1.81	0.00	1.36–2.40
	>80:1	318/636	50.0	1.55	1.07–2.22	1.50	0.03	1.05–2.15	266/691	38.5	1.14	0.75–1.73	1.10	0.48	0.84–1.45
Female Teacher (%)	<25%	467/1080	43.2	1		1			510/1249	40.8	1		1		
	25–50%	266/632	42.1	0.95	0.62–1.45	1.08	0.71	0.73–1.58	232/681	34.1	0.70	0.46–1.07	0.94	0.68	0.71–1.25
	>50%	145/351	41.3	0.95	0.57–1.58	1.90	0.00	1.26–2.88	167/448	37.3	0.77	0.48–1.24	0.91	0.54	0.67–1.23
School Rank	Low < 50%	139/276	50.4	2.06	1.36–3.13	3.04	0.00	1.61–5.74	95/208	45.7	1.50	0.86–2.61	1.43	0.16	0.87–2.34
	Mid 50–75%	177/530	33.4	1		1			231/625	37.0	1		1		
	High:>75%	562/1257	44.7	1.72	1.22–2.41	1.92	0.00	1.23–3.00	583/1545	37.7	1.21	0.78–1.89	1.33	0.08	0.96–1.82
Access to water	No	168/359	46.8	1		1			165/374	44.1	1		1		
	Yes (piped/borehole)	710/1704	41.7	0.84	0.56–1.25	1.25	0.38	0.76–2.05	744/2004	37.1	0.78	0.51–1.19	0.73	0.11	0.49–1.07

¥ Adjusted for individual, household and school-level factors.
